# Transient Quadriplegia: A Case-Based Approach to Cervical Trauma

**DOI:** 10.5811/cpcem.2020.12.49364

**Published:** 2021-02-23

**Authors:** Raymond Jabola, Benjamin Boswell, Robert H. Lutz, Jack Casey, Anthony Ceraulo

**Affiliations:** * Case Western Reserve University School of Medicine, Department of Emergency Medicine, Cleveland, Ohio; † Davidson College, Department of Sports Medicine, Davidson, North Carolina; ‡ Duke University Medical Center, Department of Orthopedics, Durham, North Carolina

**Keywords:** transient, quadriplegia, neuropraxia, cervical, trauma

## Abstract

**Introduction:**

Spinal cord injuries are a common reason for presentation to the emergency department (ED). Sports-related spinal injuries are one of the least common spinal injuries, falling behind vehicular accidents, acts of violence, and falls.

**Case Report:**

This case report describes a case of transient quadriplegia in a 17-year-old male who presented to the ED after a helmet-to-helmet collision while participating in football.

**Conclusion:**

Emergency physicians should be cognizant of potential spinal cord injury using clinical decision tools and radiologic imaging to properly disposition a patient presenting with cervical spine injury.

## INTRODUCTION

Since 2015, 7.8% of spinal cord injuries (SCI) have been attributed to a sports incident.^1^ There are approximately 17,810 new SCI cases each year in the United States (approximated population 329 million in 2020).^1^ The annual incidence of sports-related SCI is estimated to be 1389 cases per year. Because a majority of SCI cases are due to vehicular accidents, acts of violence, and falls, sports-related SCI are regarded as rare, varying in demographics and difficult to predict in healthy individuals. Patients who present in the emergency department (ED) with SCI must be properly triaged and evaluated to reduce the risk of neurological damage.

The severity of SCI can range from transient to permanent injury. On this spectrum, the least severe injury is known as neuropraxia, which is defined as a transient loss of motor or sensory function that can last from less than 15 minutes to 48 hours. Cervical cord neuropraxia occurs most commonly in contact sports, with the highest rates found in football players estimated to be 7.3 per 10,000 individuals.^2^ In this case report, cervical cord neuropraxia will be considered synonymous with transient quadriplegia (TQ). The mechanism of injury in TQ results from hyperflexion, hyperextension, or direct axial load of the cervical spine. In football injuries, this tends to occur from flexion of the cervical spine in addition to a concurrent axial force. The resulting neurological dysfunction can be anxiety-inducing to both the individual and the medical teams involved in patient care. When approaching traumatic cervical injuries, appropriate ED response is comprised of concise clinical decision-making, proper imaging studies, and appropriate consultations.

The case we present here pertains to a football player who presented to the ED with TQ. The purpose of this case report is to discuss the pathophysiology and presentation of TQ, as well as recommendations for the ED approach to SCI, particularly cervical spine injury.

## CASE REPORT

A 17-year-old Black male presented to the ED with weakness. Prior to arrival to the ED, the patient was involved in a helmet-to-helmet collision while participating in a football game. Physicians from both teams were called onto the field by the athletic trainer after the patient reported paralysis of bilateral upper and lower extremities as well as loss of sensation from the neck down. The cervical spine was manually stabilized and emergency medical services (EMS) were alerted. While the patient’s cervical spine was stabilized, his helmet and shoulder pads were removed. Over the course of 10 minutes as EMS was pending arrival, he regained sensation and movement of his extremities. After regaining movement and sensation, on-site examination demonstrated sixth cervical (C6) vertebral tenderness to palpation, but no other abnormalities. The patient’s cervical spine was manually held and he was placed onto a backboard. He was then transferred to the ED.

Ancillary information was provided indicating the patient had a similar episode two weeks prior. That episode involved a similar helmet-to-helmet collision that resulted in a sensation of “shock” in which he could not move for several seconds but was ultimately able to stand on his own. On-site physical exam was negative and without any acute physical findings. A concussion screen was performed, which was normal.

Upon ED arrival, the patient denied having a headache, change in vision, tinnitus, loss of consciousness, nausea, vomiting, confusion, chest pain, shortness of breath, back pain, or abdominal pain. He remembered the entire event and had no history of concussions. His vital signs included a blood pressure of 119/53 millimeters of mercury, pulse of 84 beats per minute, respiratory rate of 25 respirations per minute, a temperature of 100.1° Fahrenheit (37.8° Celsius), and an oxygen saturation of 100% on room air. He was oriented, well appearing, and in no acute distress. Head and cervical spine computed tomography (CT) were unremarkable, and he had no fractures.

**Image 1:**
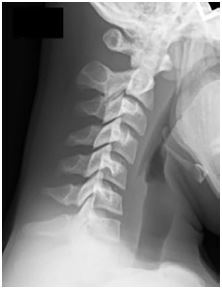
Plain lateral cervical radiograph in flexion without abnormalities.

**Image 2:**
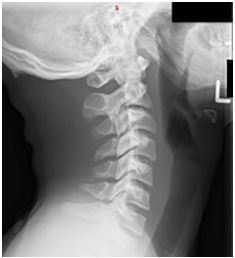
Plain lateral cervical radiograph in extension without abnormalities.

Cervical spine lateral flexion-extension radiographs did not show any cervical instability (Images 1 and 2). He ambulated normally following removal of the cervical collar and was alert with normal mood and affect. His physical exam and neurological exam were unremarkable. He had no cranial nerve deficits, and he had full strength and sensation in upper and lower extremities bilaterally. The ED determined the patient likely sustained brachial plexus neuropraxia, known in the sports medicine world as a “stinger.” He was discharged with return precautions and a follow-up appointment with sports medicine.

**Image 3:**
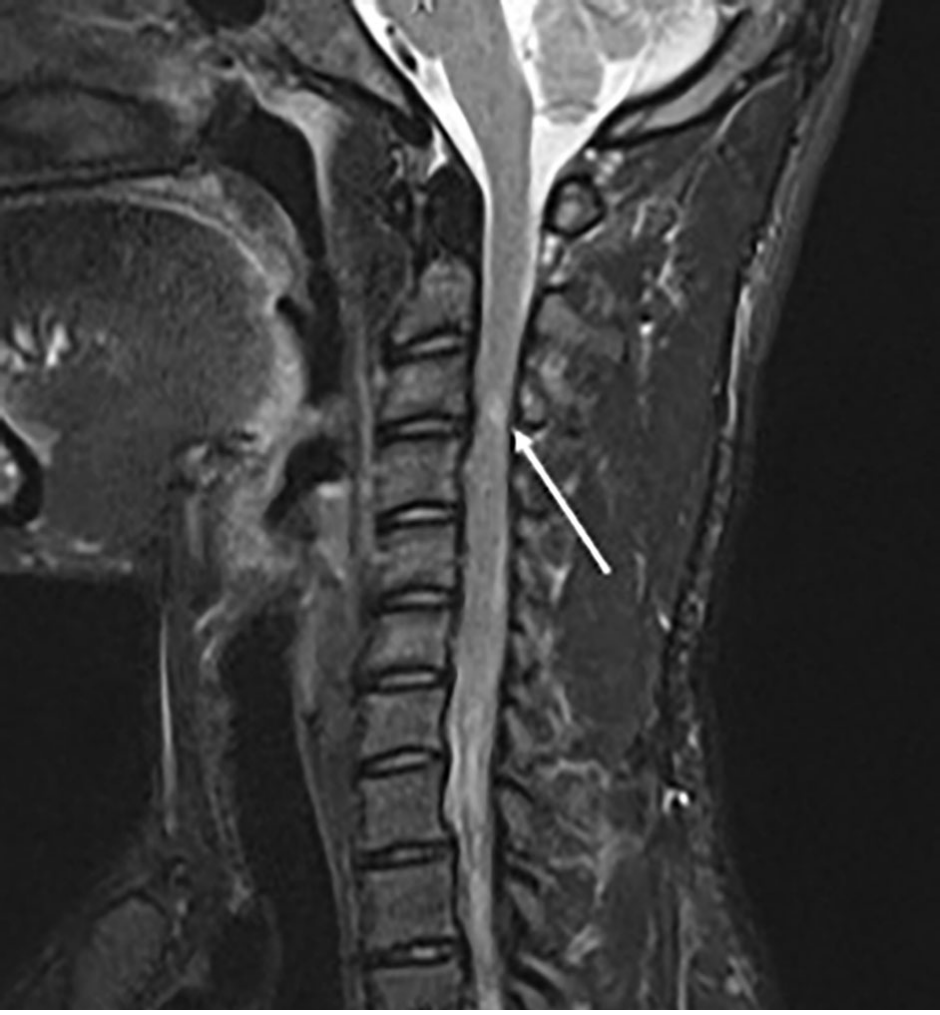
Magnetic resonance imaging with arrow demonstrating cervical spinal cord edema at the site of congenital spinal canal narrowing of 11 millimeters.

The patient followed up with sports medicine three days following the injury and was asymptomatic. Magnetic resonance imaging (MRI) was ordered, which showed congenital cervical spinal canal narrowing at C4 with a canal width of 11 millimeters (mm) (normal range 15–27 mm) (Image 3). He also had C3–C4 bilateral spinal cord contusions and a C6 bone contusion. He was referred to a spine surgeon who recommended permanent disqualification from participation in contact sports.

**CPC-EM Capsule**

What do we already know about this clinical entity?

*Transient quadriplegia has been associated with patients who have cervical stenosis and can occur in populations with no known previous medical history*.

What makes this presentation of disease reportable?

*The presentation of transient quadriplegia is important due to the potential complications from inadequate evaluation of cervical trauma*.

What is the major learning point?

*The major learning point is to discuss appropriate evaluation of patients presenting to the emergency department with concerns for cervical trauma*.

How might this improve emergency medicine practice?

*This case report is aimed to increase emergency physician awareness and discuss the approach to cervical trauma that can occur in sports related injuries*.

## DISCUSSION

The pathophysiology of TQ involves a non-neutral cervical position in addition to an axial force. In football injuries, this is usually seen in hyperflexion of the cervical spine with an axial force, which can occur during helmet-to-helmet collision. This causes pincer-like compression of the cervical spine between two vertebrae within the spinal canal. It is theorized that the compression causes a prolonged depolarization of the neural tissue, thus inhibiting further action potentials (seen in in-vitro studies).^3^ Patients at risk for TQ are those with a smaller ratio of spinal canal diameter to vertebral body diameter.^4^ This is known as the Torg-Pavlov ratio, coined after orthopedic surgeon Joseph S. Torg and radiologist Helene Pavlov who introduced the ratio method for measuring cervical cord stenosis in the 1980s.

Most patients presenting to the ED with SCI have associated injuries. According to *Advanced Trauma Life Support* (ATLS), 10^th^ ed, 55% of spinal injuries occur in the cervical region, 15% in the thoracic region, 15% in the thoracolumbar junction, and 15% in the lumbosacral junction.^5^ Therefore, in patients with high concern for spinal trauma, high precautions should be placed. Based on the ATLS trauma algorithm, primary and secondary surveys should be performed in a stepwise pattern to ensure a traumatic injury is not missed. Of patients who present with a spinal injury, 25% of these spinal injuries will have a concurrent brain injury.^5^ Hence, the disability assessment should include a Glasgow Coma Scale to trend mental status changes, and proper radiologic imaging to diagnose traumatic injuries including possible head trauma.

In patients who present to the ED with TQ, they do not commonly experience neck pain and loss of cervical range of motion around the time of injury.^6^ Approximately 74% of these patients will have a resolution of neural symptoms within 15 minutes. Only 11% will have symptoms lasting greater than 24 hours. Approximately 80% of these patients will have neural deficits in all four limbs.^7^ In patients who present to the ED, emergency physicians must determine the necessary imaging for each patient.

Two commonly used clinical decision tools help emergency physicians assess whether a patient requires cervical imaging: the National Emergency X-ray Utilization Study (NEXUS); and the Canadian Cervical Spine Rules. Incorporated in these tools are history and physical examination findings, which would indicate the need for radiologic imaging. Such findings would include midline cervical spine tenderness, paresthesia, and mechanism of action. A systematic review of the two clinical decision tools from 2012 found the sensitivity of the Canadian Cervical Spine Rules ranges from 0.90 to 1.00 with a specificity ranging from 0.01 to 0.77, and the sensitivity of the NEXUS criteria ranges from 0.83 to 1.00 with a specificity ranging from 0.02 to 0.46. Due to their high sensitivity, these decision tools are often used in the ED to rule out the need for imaging in patients with suspected cervical spine injury.^8^

In determining the best imaging study, CT of the cervical spine has become the gold standard for screening in cervical spine trauma. According to the 2009 Eastern Association for the Surgery of Trauma (EAST) practice management guidelines, the recommended primary modality of imaging of the cervical spine in those suspected to have a cervical spine injury is axial CT. Plain radiographs provide no additional information and are advised against due to low sensitivity. In a non-altered patient with negative neurological deficits, a negative CT of the cervical spine is sufficient and EAST guidelines recommend against any further imaging. Should there be a neurological deficit attributable to cervical spine injury, EAST recommends an MRI and neurosurgical consultation.^9^ The practice of using MRI in an acute traumatic setting is variable and differs from institution to institution; it is based on the select populations of interest such as obtunded patients.

Appropriate consultation should take place, whether that be in the hospital or outpatient setting. Neurosurgical spine consultation should be considered in patients with unstable injuries or those with neurological dysfunction. If a proper ED evaluation is performed with negative findings without any residual neurological deficits, a referral to a sports medicine physician would be of value to patients to discuss risks and benefits of returning to contact sports.

## CONCLUSION

Sports-related injuries are unpredictable and cause a spectrum of disability to patients. When this patient population presents to the ED, appropriate assessment, clinical decision-making, and imaging is of great importance to reduce prolonged disability. In the case discussed, the patient presented after a 10-minute episode of quadriplegia caused by helmet-to-helmet contact during a football game. After evaluation in the ED, the patient was discharged home with sports medicine follow-up, where an MRI was performed and he was found to have spinal cord contusion at C3–C4. Emergency physicians should be cognizant of potential spinal cord injury using clinical decision tools and radiologic imaging to properly disposition a patient presenting with cervical spine injury. In those patients who are diagnosed with transient quadriplegia, they will need to have an informed discussion with their sports medicine physician or neurosurgeon to reduce the risk of future morbidity.
